# Stress and Coping in Paediatric Oncology

**Published:** 1988

**Authors:** Geoffrey Moss

**Affiliations:** Senior Educational Psychologist, Avon


### Stress and coping in paediatric oncology

**Geoffrey Moss**
Senior Educational Psychologist, Avon

While the concept of stress has enjoyed increasing research attention in the psychological literature, it is also one that enjoys a wide range of definitions. At the same time, regardless of its heuristic value as a means of understanding and explaining aversive reactions to certain events, the paradigm of stress and coping has until recently received scant attention in the field of paediatric oncology (and indeed in child development research generally; see Garmezy & Rutter, 1983). For example, in a recent review of studies on anticipatory nausea and vomiting in cancer chemotherapy (Burish & Carey, 1986), out of seventeen reported studies since 1979 only one (Dolgin, Katz, McGinty & Siegel, 1985) was concerned with paediatric patients. Despite a growing accumulation of descriptive accounts of the likely stresses for paediatric oncology patients, there remains little empirical data adduced from the experiences and evidence of the children themselves.

There is general agreement that the notion of stress relates to the circumstances that make physical or psychological demands on an individual and to the emotional reactions that are experienced by the individual in that situation. Psychological stress can be defined by those transactions between the person and the environment in which stressors are linked to anxiety reactions by the perception of threat. An important feature in this is that it is the individual who determines whether or not a certain experience or event is stressful for him or her. We have to invoke the concept of cognitive appraisal. What may appear threatening and anxiety provoking to one child may not so appear to another. The extent to which any event may appear threatening will be influenced by the child's secondary appraisal of his own coping resources. The events may appear stressful but not overwhelmingly so if the child can utilise coping strategies to endure, minimise or overcome the aversive aspects of that event. Coping, then, can be defined as those efforts, both action-oriented and intrapsychic, to manage (i.e. master, tolerate, reduce, minimise) both environmental and internal demands (and conflicts that may arise between those demands) which otherwise tax or exceed a person's resources (Lazarus & Launier, 1978). Stress and coping are part of the same interaction. Those elements which are construed as providing some buffering effect upon a stressor (e.g. personality factors, cognitive style, social support) are also enhancing the coping response.

A study currently in progress at Bristol Children's Hospital is looking at what features of the illness, treatment and hospital experience are perceived as stressful by paediatric oncology patients, and what sorts of coping responses are effective in reducing the stress of cancer treatment. Children aged from 7 to 16 years admitted to the Paediatric Oncology Department since January 1986 form the population for this research, to provide a prospective longitudinal study. Shortly after admission they complete a series of personality questionnaires and structured interview schedules, together with background data from their parents and schools. During the course of their treatment observation schedules of children's behavioural responses related to mood states are completed by nursing staff, supplemented by the informal observations of parents and patients themselves as appropriate. Follow-up interviews of parent, patient, nursing and medical staff are completed after six months from diagnosis. In the first eight months of the study 18 children aged 7 to 16 years were admitted, and a preliminary analysis of the information is here reported on 13 for whom 6 months have elapsed since diagnosis.

The most frequently cited stressful event was related to invasive medical procedures. Of the 13 patients, 8 had their chemotherapy treatment by intravenous drug administration through a catheter, and it is this that caused most distress for the majority of them. Some patients were admitted with an existing aversion to needles, acquired from previous dental or medical experiences. For them the anxiety and pain associated with needles became a major problem of coping as treatment progressed. Apart from one patient, there were none for whom the stresses of i.v. needle procedures had not increased by the end of six months' treatment. The children's own account of this was typically that their veins became increasingly difficult to find in order for a cannula to be inserted, and also tended to 'tissue' so that the needle had to be resited. Children's coping resources are thus overtaxed by repeated i.v. needle procedures; no stable pattern of habituation seems to occur. Early successful coping with needles does not predict later coping; early difficulties in coping do predict increasing problems later. The insertion of a "long line" (a semi-permanent Hickman catheter through which i.v. drug treatment can be given) was perceived by patients who had had some of their treatment without it as a definite benefit in reducing anxiety associated with treatment.

Ways of coping with needles showed little difference among all the children, even comparing the 'better copers' with the 'worse copers' in terms of needle stress. They all tended to tense their bodies, grab hold of something to squeeze or to bite on. The better copers, however, were able to look away from the needle, and consciously attempted some form of distraction, visual or auditory. One described how he might put a finger in his mouth to bite on it: "Causing a bit of pain elsewhere takes away attention from my vein, and I can control the amount of pain I give my finger". In contrast the worse copers gave all their attenton to the needle, and used no form of distraction.

Vomiting as a result of chemotherapy was the next most common stressor. Ways of coping with this varied. Some children welcomed the sedative effect of anti-emetics and used sleep as a psychological anaesthetic, reporting after each chemotherapy cycle little recall or bother about sickness. Other children, conversely, did not like the feeling of drowsiness and, as they perceived it, consequent loss of control. Some felt they could exercise more control over nausea and vomiting by remaining alert, consciously relaxing stomach muscles, breathing gently, and then focusing mental attention on something else. With one exception, all those children who had reported some upset caused by chemotherapy nausea found their vomiting becoming less of a bother by the end of the six month period. They also had begun to form their own assessment of which chemotherapy drugs were most upsetting and which anti-emetics were more or less useful to them.

Concerns about losing hair were expressed by 6 out of 8 girls and by none of the boys. When they were first told about treatment and its possible side-effects, it was the news of hair loss that was often the trigger for tearful reactions. The distress proved to be more in the anticipation than in the event. Those girls who were receiving frequent inpatient treatment and were often unwell were relatively less concerned about their appearance than those who were able to resume school attendance. None of the girls attended school without a wig or headscarf, and fear of discovery has been a mild cause of anxiety for some. The same concerns have not been as evident among the boys, although the older tend to conceal their baldness more frequently than the younger. Conformity, a desire to fit in with peers, not to appear different, seems to govern behaviour in these cases.

Admission to hospital was stressful for the majority of the children initially, but most found the positive atmosphere on the ward allayed their anxieties. Subsequent inpatient attendance at hospital was either associated with treatment, or with missing school and family. Children with academic aspirations were concerned about falling behind with their studies. What they viewed as additional 'non-treatment' hospital admissions (e.g. because of post-treatment neutropenia and infection), caused them additional anxiety. They tried to keep up with school work by bringing books and assignments into the ward, and doing extra study afterwards to catch up. In addition children became concerned about losing out on friendships, that while in hospital for long periods they would become "out of it" and not have access to their old circle of friends on return to school. Some reported that the friendships they made with nurses compensated for this, particularly some teenagers for whom hospital became as much a social as a medical environment. Inevitably most of the younger patients missed being with their families, and the presence of one or both parents on the ward most of the day proved an important source of security.

Probably the most pervasive, and yet most difficult stress to quantify, has been the anxiety of having a potentially fatal illness. Many of the older children were able to acknowledge that when first told the nature of their illness they feared the worst. Not many are familiar with the terminology of their illness initially, but through formal and informal sources they rapidly acquire a working understanding of it. Some can cope with the awareness that theirs is a potentially terminal condition, and then of the need to think positively and not dwell on negative thoughts. Others translate the information that it *can* be cured to it *will* be cured. None of the children in this preliminary study displayed pessimistic hopelessness in the face of their illness. Their denial or avoidance of the grim possibilities seemed quite appropriate coping strategies. They all accepted the need to undergo all the stresses so far referred to in the way of treatment, yet most of the time otherwise remained hopeful and positive. As one fifteen year old boy put it "I'm not particularly scared of the consequences of a relapse, but sometimes when I get a temperature for no apparent reason I might think 'What's happening, have I relapsed?' but otherwise I've just got to assume that everything is O.K., that at the end of two years treatment I'll be O.K. If I just worried all the time, then I'd not get any benefit from being in remission now."

Other stressors reported tend to be more specific to the child's own type and progress of treatment. Uncertainty about future treatment created anxiety, particularly where there was the possibility of surgery. For one boy with a bone tumour the possibility of amputation remained his major and constant source of anxiety until surgery was completed, after which he enjoyed a brief period of relief until focusing on his next concern. For him it was important to have something to worry about, as if that provided a reassurance that if some negative event should occur he was emotionally prepared for it. Such a coping style was utilised at the cost of his interpersonal relationships.

In assessing whether coping responses are appropriate we have to ask 'appropriate for what?'. There may be costs and benefits in many coping strategies depending upon the circumstances in which they are deployed. (Lazarus, 1981). In assessing which children proved to be better or worse copers with the stresses of illness and treatment we might judge how effectively they were able to minimise or master each stressor, maintain a reasonable level of self-esteem, and maintain reasonable relationships with family, friends, medical and nursing staff, depending upon their environmental circumstances. Using preliminary measures of adjustment in this way allows us to identify a group of 'better copers' to compare with a group of 'worse copers', and then to see what differences, if any, appear between the groups.

Better copers tended to be different in terms of personality. They were stable, calm, self-controlled individuals, gaining low scores on measures of neuroticism and anxiety. Extroversion/introversion factors were not different. Worse copers were more overreactive, displaying more anxiety on admission to hospital. Better copers tended to be achievement-orientated. They were more academically successful at school, compared to the worse copers, two of whom had needed special help in school because of learning problems. Perhaps related to this factor, the better copers tended to be more creative in generating flexible strategies for dealing with problems. They were more independent and self-reliant, but at the same time able to make positive use of coping suggestions from other sources. They made extensive use of positive self-reassurance and optimistic thinking, tending to view their illness as a challenge that would not beat them. In terms of their previous histories, the better copers did not recall any negative hospital or medical experiences, whereas the worse copers already had anxieties about hospital or aversions to invasive medical procedures.

There were differences in terms of medical factors as well as personality factors. Although there were no differences in the predicted toxicity of treatment between the groups, the worse copers spend significantly longer periods in hospital and there were more deviations from the original treatment plan[1]. Worse copers had a poorer prognosis for recovery from the onset, and experienced more setbacks in their treatment. Among the good copers all completed the planned cycles of chemotherapy without a Hickman catheter. Among the worse copers one began treatment with a Hickman inserted, a second had one inserted in the early part of treatment, and the other two have since had them inserted.

It seems tempting to assume that poor prognosis, lengthy hospitalisation and deviation from treatment plan would explain the observed differences between the groups in terms of the increased stress thus placed upon the one, with a consequent overburdening of coping resources.* However, differences in coping competence began to emerge early on in treatment, when treatment and hospitalisation differences between the groups were not established. Perhaps it ncould be argued that psycho logical factors influenced medical outcomes, as well as the reverse. We would need to compare groups of good and poor copers having the same medical prognosis to do this. That has not yet been possible with this study.

* Predicted toxicity of chemotherapy and length of time spent in hospital were significantly related for this study group overall. p 0.02.

There are other factors that relate to stress and coping not dealt with here: hospital environment, staff support, family support and coping etc. which may all interact to alter the child's experience of stress. It is also clear that there are differences between the stresses reported in this study and those in the U.S. literature. Studies in the U.S.A. point to recurring problems children there face in coping with bone marrow aspirations an lumbar punctures. None of the patients undergoing these procedures with local anaesthetic in Bristol reported any particuler distress. Again, U.S. studies point to the problems of anticipatory nausea and vomiting. It is estimated that about 20% of children receiving chemotherapy will develop anticipatory vomiting (Dolgin et al, cited above). Several intervention studies have been reported whose aim was to reduce nausea and vomiting (e.g. Zelter, Le Baron & Zelter 1984). None of the children in our Bristol study has developed anticipatory nausea or vomiting as a conditional response to chemotherapy.

All this points to a constellation of features that influence the experience of stress for children undergoing cancer treatment: at a medical level—the type of treatment regime, the medical procedures employed; at a social level—the supportive relationships between staff and family, the coping resources of parents in supporting their children; at the individual level—dispositional attributes in the child and personal resilience, that allow for the effective deployment of appropriate coping strategies.

### REFERENCES

BURISH, T. G. and CAREY, M. P. (1986) Conditioned Aversive Responses in Cancer Chemotherapy Patients: Theoretical & Developmental Analysis. *Journal of Consulting and Clinical Psychology*, **54**, **5**, 593–600.

DOLGIN, M. J., KATZ, E.R., McGINTY, K. and SIEGEL, S. (1985) Anticipatory Nausea and Vomiting in Paediatric Cancer Patients. *Paediatrics*, **75**, 547–552.

GARMEZY, N. and RUTTER, M. (Eds.) (1983) *Stress, Coping and Development in Children.* N. Y. McCraw-Hill.

LAZARUS, R. S. (1981) The Cost and Benefits of Denial. In Dohrenwend, B. S. and Dohenwend, B. P. (Eds.). *Stressful Life Events and Their Contexts* N. Y. Prodist.

LAZARUS, R. S. and LAUNIER, R. (1978) Person and Environment. In Perrin, L. A. and Lewis, M. (Eds.). *Perspectives in Interactional Psychology.* N. Y. Plenum Press.

ZELTZER, L. and LeBARON, S. (1983). Behavioural Intervention for Children and Adolescents with Cancer. *Behavioural Medicine Update,* **5**, **2/3**, 17–22.

ZELTZER, L., LeBARON, S. and ZELTZER, P. (1984) The Effectiveness of Behavioural Intervention for Reduction of Nausea and Vomiting in Children and Adolescents receiving Chemotherapy. *Journal of Clinical Oncology,* **2**, **6**, 683–690.

